# Long QT interval and syncope after a single dose of COVID-19 vaccination: a case report

**DOI:** 10.11604/pamj.2021.40.67.31546

**Published:** 2021-09-30

**Authors:** Nahid Azdaki, Marjan Farzad

**Affiliations:** 1 Cardiovascular Diseases Research Center, Department of Cardiology, School of Medicine, Birjand University of Medical Sciences, Birjand, Iran,; 2 Clinical Research Development Unit of Razi Hospital, Birjand University of Medical Sciences, Birjand, Iran,; 3 Cardiovascular Diseases Research Center, School of Nursing and Midwifery, Birjand University of Medical Sciences, Birjand, Iran

**Keywords:** COVID-19, long QT interval, case report

## Abstract

Adverse consequences of the coronavirus disease 2019 (COVID-19) vaccination which have been reported in scientific papers are varied. One possible but rare consequence is myocarditis, which may have a diversity of clinical manifestations. We report a case of a 70-year-old man who presented to the hospital for some syncope, 3 days after his first COVID-19 AstraZeneca Vaccination. Initial electrocardiogram (ECG) showed a long QT interval (QTc = 600 milliseconds). Laboratory tests revealed elevated troponin and lack of evidence of viral infection. Further investigations revealed the vaccine-induced myocarditis and arrhythmias linked to it. Within one week of magnesium treatment, the QT interval was completely corrected, and the patient discharged with no typical syncope attacks. This case like the previous reported one confirms that myocarditis is a complication of COVID-19 vaccine, but implies its clinical manifestations may be varied and even may happen after the single dose of vaccination.

## Introduction

The coronavirus disease 2019 (COVID-19) has spread rapidly into a pandemic. Vaccination is a well-approved part of a preventive schedule, but is not without side effects. Minor complications such as pain, redness and swelling at the injection site and systemic symptoms of headache, fatigue, muscle pain, fever and chills have commonly been reported [1]. Myocarditis is a serious complication which has been reported for Pfizer-BioNTech and Moderna vaccine in several reports [2, 3], range from mild asymptomatic inflammation of the heart to severe heart failure and death [4].

## Patient and observation

**Patient information:** a 70-year-old man with medical history of hypertension (HTN) and diabetes mellitus under medical treatment presented to the emergency department for 1-2-minutes consecutive syncope attacks, 3 days after his first COVID-19 AstraZeneca Vaccination.

**Clinical findings:** due to facial injury in the first episode of the syncope, a brain CT was performed to rule out the possibility of head trauma, and the brain CT was normal. Any symptoms of chest pain or dyspnea were not reported by the patient, and both vital signs (HR = 63, BP = 110/70, T = 36.5, RR = 14) and physical examination were normal.

**Figure 1:**
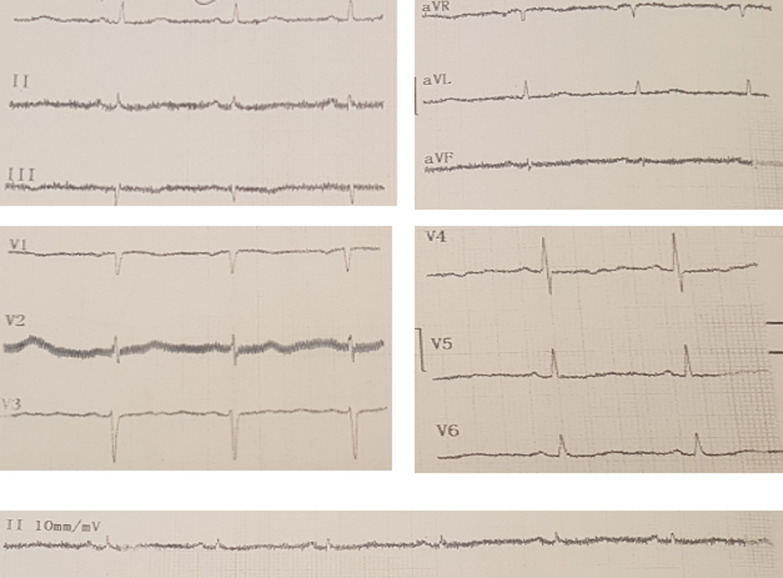
normal sinus rhythm, normal axis deviation, no significant ST-t changes, long QTc interval: QTc = 600ms

**Figure 2:**
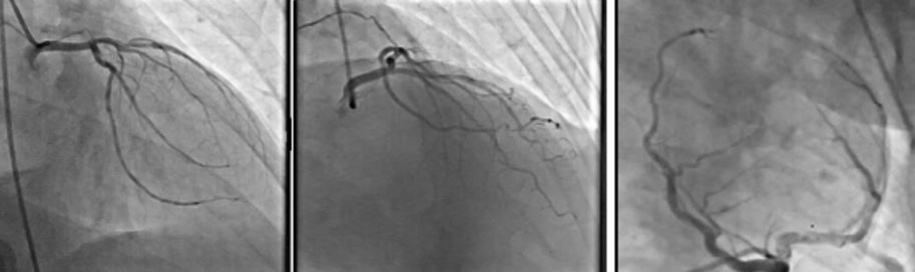
coronary angiogram revealed normal coronary arteries

**Diagnostic assessment:** the long corrected QT interval (QTc = 600ms) and a sinus rhythm without ischemic changes were seen in the Initial ECG (Figure 1). Laboratory studies showed: elevated troponin I (1.1ng/mL, normal 0.01-0.04), elevated C-reactive protein (20mg/L, normal < 10.0) and negative polymerase chain reaction (PCR) for COVID-19. A transthoracic echocardiogram was within normal limits, and the left ventricular ejection fraction (LVEF) was 60%. No wall motion abnormality was detected. The mild pleural effusion, which was reported in the patient's high-resolution computed tomography (HRCT), was not verified through the transthoracic echocardiogram or other clinical findings. Coronary angiography revealed no significant coronary artery disease (CAD) (Figure 2).

**Therapeutic intervention:** the patient underwent magnesium sulfate treatment (2g IV q4h, cardiac monitoring and blood potassium and magnesium modification for the possibility of myocarditis and the arrhythmias linked to it due to the long QTc and K = 3.4meq/L (normal 3.5-6meq/L).

**Figure 3:**
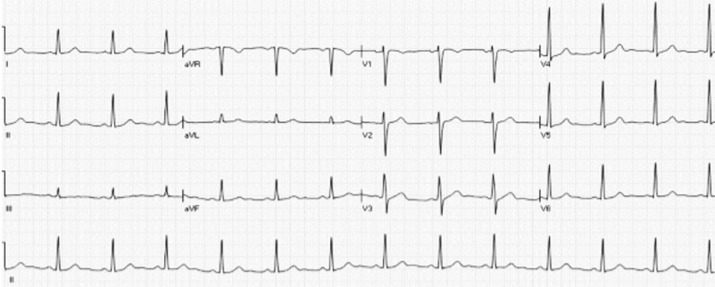
normal sinus rhythm, normal axis deviation, no significant ST-t changes, normal QT interval

**Follow-up and outcomes:** four hours later, the long QTc began to correct, and potassium was in the normal limit. No typical syncope attack was reported in the patient. Within one week the QT interval was completely corrected (Figure 3), and both troponin I and CRP were reduced.

## Discussion

Vaccination is a well-approved part of a preventive schedule, but is not without side effects. Among the mRNA vaccines, myocarditis is a serious but rare complication which has been reported for Pfizer-BioNTech and Moderna vaccine in several reports [2, 3] and contrary to our report, the most cases occurred after the second dose of vaccination [5]. As reported by the US Centres for Disease Control and Prevention (CDC), the rates of myocarditis are 12.6 cases per million doses of second-dose COVID-19 vaccine among young males aged 12-39 years [6]. Viral infection is usually the main etiology of myocarditis. In this case, the negative PCR test for COVID-19 and viral serology may be signalling the possibility of vaccine-related myocarditis.

Clinical symptoms, laboratory findings, electrocardiographic and echocardiographic parameters are the common diagnostic features. Clinical presentation of COVID-19 myocarditis varies among cases. Some patients present mild symptoms like fatigue and dyspnoea [7] while others report chest pain [8]. Many patients show acute heart failure [8]. Blood tests in myocarditis patients show elevated levels of inflammatory markers like C-reactive protein and cardiac enzyme like troponin I [9]. EKG abnormalities may include QT prolongation or pseudo infarct pattern [10]. Depressed left ventricular ejection fraction (LVEF) may be reported based on the patient's echocardiography [10]. In this case, the only abnormalities we observed were long corrected QT interval (QTc=600ms) in the electrocardiogram and enzymatic raise, accompanied by syncope attacks. With regard to raised cardiac troponin levels, epicardial disease was ruled out by coronary angiography. However, cardiac Magnetic resonance imaging (MRI) could be helpful to fulfil the criteria for myocarditis in patients with suspected myocarditis [3].

Long QTc interval is a good predictor of myocarditis and is useful for early recognition of the fulminant one [10]. QTc > 500ms is associated with an increased risk factor of *torsade de pointes*. The rhythm may terminate spontaneously, presenting syncope attack, or may degenerate into ventricular fibrillation [10]. In this case, syncope attacks were related to arrhythmias linked to myocarditis and subsequent electrocardiographic changes.

## Conclusion

Although myocarditis has been reported following COVID-19 vaccination, especially in double-jabbed people, further research will be needed to verify its occurrence after a single dose of COVID-19 vaccination. Furthermore, the diversity of clinical manifestations of myocarditis in COVID-19 vaccinated people should be taken into account by the clinician.

## References

[1] Center for Disease Control and Prevention (CDC) Possible Side Effects After Getting a COVID-19 Vaccine.

[2] Bruce Y Lee, Are Rare Cases Of Myocarditis Linked To Pfizer, Moderna COVID-19 Vaccines?.

[3] Albert E, Aurigemma G, Saucedo J (2021). Myocarditis following COVID-19 vaccination. Radiology Case Reports.

[4] Fung G, Luo H, Qiu Y, Yang D, McManus B (2016). Myocarditis. Circulation Research.

[5] Staff Toi Israel said probing link between Pfizer shot and heart problem in men under 30.

[6] Bozkurt B, Kamat I, Hotez PJ (2021). Myocarditis With COVID-19 mRNA Vaccines. Circulation.

[7] Kim IC, Kim JY, Kim HA, Han S (2020). COVID-19-related myocarditis in a 21-year-old female patient. Eur Heart J.

[8] Zeng JH, Liu YX, Yuan J, Wang FX, Wu WB, Li JX (2020). First case of COVID-19 complicated with fulminant myocarditis: a case report and insights. Infection.

[9] Inciardi RM, Lupi L, Zaccone G, Italia L, Raffo M, Tomasoni D (2020). Cardiac Involvement in a Patient With Coronavirus Disease 2019 (COVID-19). JAMA Cardiol.

[10] Caforio ALP, Pankuweit S, Arbustini E, Basso C, Gimeno-Blanes J, Felix SB (2013). Current state of knowledge on aetiology, diagnosis, management, and therapy of myocarditis: a position statement of the European Society of Cardiology Working Group on Myocardial and Pericardial Diseases. Eur Heart J.

